# How has the tobacco industry passed tax changes through to consumers in 12 sub-Saharan African countries?

**DOI:** 10.1136/tc-2023-058054

**Published:** 2023-08-11

**Authors:** Zaineb Danish Sheikh, J Robert Branston, Kirsten van der Zee, Anna B Gilmore

**Affiliations:** 1 Department of Health, University of Bath, Bath, UK; 2 School of Management, University of Bath, Bath, UK; 3 School of Economics, University of Cape Town, Rondebosch, Western Cape, South Africa

**Keywords:** Low/Middle income country, Price, Taxation, Tobacco industry

## Abstract

**Introduction:**

Tobacco taxation is only effective in reducing consumption when it is translated into higher prices. This study aims to investigate to what extent the tobacco industry (TI) passes tax increases over to consumers by increasing the retail price of cigarettes in 12 sub-Saharan African (SSA) countries.

**Methods:**

African Cigarette Prices Project and WHO’s Global Tobacco Epidemic Reports data were used to calculate the rate of tax pass-through by decomposing the retail price of cigarettes into tax and net prices between 2016 and 2020. Percentage change in net price was used to identify industry pricing behaviour, in both packs and single-stick sales. TI pricing strategies were examined by country, producer type, producers, and cigarette price segment.

**Results:**

There were mixed TI strategies, with taxes primarily overshifted (Botswana, Madagascar, Tanzania, Zimbabwe), undershifted (Ethiopia, Lesotho, Mozambique, Namibia, South Africa, Zambia) or a mix of both (Malawi, Nigeria). The detail varied between countries, over time, and between the different brands/segments offered. Patterns for single-stick sales were broadly similar to that of packs but with some differences observed in particular countries/years. Pricing strategies for the various transnational tobacco companies and domestic producers were similar but the changes in net price for the latter were larger. The country tax level/type and the size of tax change did not seem to be an obvious influence.

**Conclusion:**

This paper provides an overview of TI pricing strategies in response to tax increases in SSA. Governments must monitor how the TI responds to tax changes to ensure that tax increases are effective in impacting price.

**WHAT IS ALREADY KNOWN ON THIS TOPIC**

- It is becoming increasingly evident that transnational tobacco companies undermine the public health impacts of increased tobacco excise taxes by adopting targeted pricing strategies to dilute their effects.
- The majority of this empirical evidence comes from high-income economies.
- Relatively less is known about strategies used across low-income and middle-income countries, especially in sub-Saharan Africa (SSA) where tobacco taxes are often low and the sale of single-sticks is often common.

**WHAT THIS STUDY ADDS**

- We explored how cigarette prices changed in response to tax increases between 2016 and 2020 in 12 SSA countries using cross-sectional local and WHO regional data, including pricing of both packs and single-sticks.
- We gathered important evidence on tobacco industry pricing strategies in response to tax increases in these countries.

**HOW THIS STUDY MIGHT AFFECT RESEARCH, PRACTICE OR POLICY**

- There is a paucity of data on tobacco pricing strategies in response to tax increases for African countries, and hence, a critical need to understand TI pricing strategies, particularly for single cigarettes, to inform effective tobacco taxation reform.
- The paper identifies industry practices, makes policy recommendations, and suggests that governments monitor cigarette prices by brand, presentation (single-sticks or packs) and price segment, and hence highlights the need to consider industry pricing strategies when setting tobacco taxes and wider tobacco control policies.

## Introduction

 Tobacco is the leading cause of preventable death worldwide, killing approximately half of its long-term users.^1^ Tobacco-attributable deaths are projected to decline in high-income countries (HICs) in the next decade, but in low-income and middle-income countries (LMICs) they are expected to double,^2^ especially in Africa where tobacco control is often relatively a low priority.^3 4^ While smoking prevalence in the African region^5^ is the lowest among all the WHO regions, a significant upsurge in the total number of smokers is predicted due to rapid expected population growth and economic development.^6 7^ The relatively high prevalence (19.1%) of tobacco use among school-going adolescents in the region,^8^ with an early average age of smoking initiation as early as <7 years,^9^ and the targeted aggressive marketing/promotional activities of the tobacco industry (TI) are further going to contribute to this predicament.^10^ Moreover, while 44 countries in the region ratified the WHO Framework Convention on Tobacco Control (FCTC), implementation and enforcement of tobacco control policies have been huge challenges for the countries in the region.

Tobacco taxation is known to be a highly effective tobacco control policy as it reduces demand for tobacco by increasing prices.^11^ It is also an efficient way of generating government revenues. WHO FCTC Article 6 recommends that taxes represent 75% or more of cigarette retail prices.^12^ However, taxation in Africa shows very slow progress towards this, with all taxes making up 40.7% of retail prices, on average; the African region ranks lowest on the Tobacconomics ‘Cigarette Tax Scorecard’ (scoring 1.64 out of 5), which assesses countries’ cigarette tax policies relative to best practices.^13^ Because taxes are so low, cigarette prices in the region are among the lowest in the world.^13^ There is also a wide variation in tax levels and structures (specific, ad valorem, or mixed) across the region, which has resulted in price variation across countries and between brands.

A growing body of evidence covering both HICs and LMICs suggests that the TI reduces the effectiveness of tobacco taxation by employing targeted pricing strategies.^14^,^18^ Differential tax shifting is one such policy, whereby the TI does not perfectly pass a tax increase on to customers, but instead shifts the tax differentially, for example, by region or brand segment. As consumers of premium products are typically less price-sensitive (are willing to pay higher prices), the industry might raise prices for premium brands by more than the tax increase, that is, overshift the tax, to maximise profits.^19^,^23^ Alternatively, for cheaper brands (whose customers are more price-sensitive) the industry may absorb tax increases (to some extent), that is, undershift the tax, to lessen the resulting price increase^24^,^27^ and minimise the reduction in demand.^28^ Existing studies have explored these tactics with relation to pack sales,^29^,^31^ but there is limited evidence in relation to single-sticks sales. One study, from Colombia, explored industry pricing for both packs and single-sticks, finding that while taxes on packs were undershifted, they were overshifted for single-sticks.^32^ This suggests a need to better understand the pricing strategies for single-sticks, as the two presentations could offer the TI more opportunities to deploy tax undermining strategies. Moreover, to date the authors are aware of no studies that explore whether pricing behaviours of domestic and transnational tobacco companies (TTCs) differ. Such knowledge is essential for effective tax policy development.

The marketing and sale of single-sticks is a significant issue in sub-Saharan Africa (SSA), even in countries where it is theoretically prohibited, such as Ethiopia, Nigeria, and South Africa (SA).^33 34^ Single-stick sales lessen the effectiveness of tax increases as they obviate the need to buy an entire pack.^35 36^ This is of particular importance for youth smoking, as such individuals are typically highly price-sensitive, and evidence shows single-sticks are often readily available to children and frequently sold near schools.^37 38^

There are currently not many independent academic studies exploring TI price-based responses to tax changes in SSA countries. This study examines how the industry passes taxes through to consumers (tax pass-through) for both packs and single-sticks, for 12 countries in SSA. It also estimates pass-through for individual tobacco companies, as well as estimating pass-through at the producer level (domestic vs TTC).

## Methodology

### Cigarette prices

We used the most recent African Cigarette Prices (ACP) Project data (2016–2020), obtained from the Data on Aliments, Tobacco and Alcohol in Africa Project, funded by the African Capacity Building Foundation and conducted by the Research Unit on the Economics of Excisable Products (REEP) based at the University of Cape Town—see box 1.^39^ At the time of the analysis there were nine publicly available rounds of the data, spanning between 2016 and 2020, from 18 SSA countries,^39 40^ all collected before the COVID-19 pandemic. To assess pass-through behaviour in a country, we need to have at least 2 consecutive years of price data to calculate the change in price following a change in tax. For this reason, we included only 12 of the 18 countries available in the ACP, which met this requirement. The included countries are: Botswana, Ethiopia, Lesotho, Madagascar, Malawi, Mozambique, Namibia, Nigeria, SA, Tanzania, Zambia, and Zimbabwe. Tax and price data were available for all the years between 2016 and 2020 for five countries: Botswana, Namibia, Lesotho, SA, and Zimbabwe. The rest of the countries had data for 4 (Malawi, Tanzania), 3 (Nigeria, Zambia), and 2 (Mozambique, Madagascar, Ethiopia) years. For simplicity, we only used price data for packs of 20 and single-sticks, because of a lack of consistent information on other packs sizes.

Box 1 **Background on the ACP project**

The African Cigarette Prices (ACP) project employs African UCT students to collect tobacco prices from their home countries during university vacation periods. The data are collected twice a year (July/August and December/January), from multiples outlets/locations, and for all brands available. Fieldworkers record the following:

- Retail price, brand/brand variant, tobacco product type, and quantity;
- Type of retailer—formal retail outlet, informal outlet, or street vendors;
- Location;
- Photographs for validation.

Details of the study protocols have been described previously. While the ACP project collects prices for various types of tobacco products, for practicality we limit our analysis to cigarettes only.

### Tobacco tax rates

Due to a lack of reliable taxation data for most of these countries, we used tax rates from the WHO’s Global Tobacco Epidemic Reports (GTR) between 2016 and 2020, which includes tax share of the retail price of a pack of 20 of the most popular brand in that country. The tax policy information is collected by the WHO regional data collectors from each country’s ministries of finance and includes data on excise taxes, value added/sales taxes, import duties, and any other taxes levied on cigarettes. In all cases, the specific brand utilised remained the same, except for the case of Mozambique from 2018, which changed to use a more expensive brand (which had no implications given there was 100% specific taxation). The GTR tax data are only available in 2 yearly intervals, therefore we used linear interpolation to estimate tax values for years not covered. Where possible, these estimates were verified by other tax sources (eg, official government sources), namely: SA, Lesotho, Namibia, Botswana, Zambia, Ethiopia, and Nigeria, and no substantial differences were identified. The percentage change in real (inflation adjusted) taxes, year to year, was calculated (see online supplemental appendix tables 1–3 for detailed information). We also used the GTR prices for the most sold brand (20 pack) for every country (in US$) to verify the ACP data prices (see online supplemental appendix table 4 for details).

### Analysis

Prices were analysed to determine whether tax changes were perfectly shifted, or differentially shifted that is, overshifted or undershifted. In the case of the tax being perfectly shifted, a US$1 increase in tax would result in a US$1 increase in retail price; with overshifting, a US$1 tax increase would result in a more than US$1 increase in retail price; with undershifting, a US$1 tax increase would result in a less than US$1 increase in retail price.

Univariate descriptive analysis using SPSS V.26 was used to characterise overall trends. TI pricing strategies were differentiated according to producer, producer type (domestic, TTC, and a small number of illicit), and price segment. Brands were allocated to one of three price segments: value (cheapest), popular, or premium (most expensive), based on the weighted average price tertiles following the WHO’s approach.^41 42^ All the available brands were allocated to a price segment based on their price over the whole period of the study, and the most popular brands in each segment were identified for analysis for each producer (TTC or domestic) based on the data frequency within the survey. These choices were verified by referencing Euromonitor country profiles and were found to be consistent in all cases. This price-based segmentation was done separately for packs and single-sticks (although in practice the allocations were consistent across packs and sticks, with only a few exceptions).

We calculated the median and mean prices for all brands, for both packs and single-sticks, collected between July and August each year for each country (January in 2020). The median was used as our estimate for price, as the mean was often skewed by outliers. All prices are reported in local currency and were adjusted for inflation by using the World Bank’s consumer price index for each country, with 2018 as the base-year (as the mid-point of the data collection period and because most countries have data available for 2018). Thus, real prices are presented except where otherwise stated. We calculated the tax paid per pack (excise—specific and ad valorem—plus VAT/sales tax) for each brand based on its median selling price. Median net price (the industry’s earnings from sales once all taxes have been paid) per pack was then calculated by subtracting the total tax paid from the median price. For single-sticks, we divided the total tax by 20 to calculate the tax per stick and then followed the same methodology to calculate net price per stick.

In line with previous studies in this area,^15 24 31 43 44^ we assess the tax pass-through by exploring changes in net price. For each brand segment and for comparability across the 12 countries (given different currencies are used), the percentage change in median net price was calculated for each year. Zero percentage change in net price over a year means perfect tax pass-through (ie, retail price changed only by the size of the tax change), a positive change means overshifting (retail price went up by more than the tax), while a negative change signifies undershifting (retail price went up by less than the tax). To provide context for the change in net price, for each year the percentage change in real tax was also calculated. Since our data cover retail prices only, we are unable to distinguish between the wholesale pricing behaviours of the TI and the retailer/distributor pricing strategies. This is particularly pertinent for the sale of single-sticks. Furthermore, for simplicity, we treated all sales of loose sticks as if they were duty-paid sales because a significant part of this market is the resale of legal, duty-paid cigarettes bought in multistick packs and which would therefore be impacted by tax changes. Likewise, all pack sales were also treated as if they were legal, duty-paid sales because (except for Ethiopia, discussed below) we had no means to identify otherwise as retail prices exceeded the tax payable.

## Results

### Tax pass-through for packs

Table 1 shows the percentage change in real net price of packs between 2017 and 2020 categorised by price, brands, producer type (TTC or domestic), and producer name. We also include the Tobacconomics Score, to provide some tobacco taxation context in each country. The results of the study illustrate there was a mixed pattern of tax pass-through, with taxes both overshifted and undershifted, and with the detail varying between countries, over time, and indeed between the different brands/segments offered.

**Table 1:** Year-to-year percentage change in real net price (2017–2020), by brand category, producers (TTC or domestic) and companies, for cigarette packs

Countries	Tobacconomics tax scores	Brand segment	Brand name	Owned by TTC or domestic company	Tobacco company	Percentage change in real tax				Percentage change in net price			
						2017	2018	2019	2020*	2017	2018	2019	2020
Botswana	4.13	Value	Pacific Blue	Domestic	PCC	9.5	4.3	−17.3	43.8	16.6	9.2	20.2	−66
		Popular	Chesterfield Blue	TTC	JTI	5.4	3.0	−25.8	59.7	−2.5	5.5	28.7	−37.1
			Craven A Menthol	TTC	BAT	6.6	2.0	−26.7	60.3	1.9	1.9	14.8	−31.3
			Rothmans Blue	TTC	BAT	6.2	0.3	−26.4	59.3	0.8	−4.5	24.9	−38.7
		Premium	Marlboro Blue Ice	TTC	PMI	10.3	2.3	−30.6	70.4	15.5	4.6	23.3	−25.3
			Dunhill Menthol	TTC	BAT	6.2	3.4	−30.4	67.4	1.3	8.4	20.3	−30
			Camel Classic	TTC	JTI	6.0	2.4	−35.2	75.0	1.8	6.2	22.4	−31.5
Ethiopia	1.50	Value	Nyala Filter	Domestic	NTE	N/A	N/A	N/A	96.9	N/A	N/A	N/A	−73.4
			Business Royals	Illicit	ITC	N/A	N/A	N/A	254.0	N/A	N/A	N/A	12.4
		Popular	Benson & Hedges Filter	Illicit	BAT	N/A	N/A	N/A	168.0	N/A	N/A	N/A	27.5
			Winston Blue	TTC	JTI/NTE	N/A	N/A	N/A	84.7	N/A	N/A	N/A	−23.4
			Rothmans Blue	TTC	BAT/NTE	N/A	N/A	N/A	35.5	N/A	N/A	N/A	−30
		Premium	Marlboro Red	TTC	PMI/NTE	N/A	N/A	N/A	21.9	N/A	N/A	N/A	−35.1
Lesotho	2.38	Value	Sun White	Domestic	Sun Tobacco	13.2	2.7	1.6	0.3	−53.8	21.1	16.6	−6.4
		Popular	Peter Stuyvesant Filter	TTC	BAT	15.0	−1.3	1.7	−0.3	1.5	−22.8	6.1	−6.6
			Camel Activate	TTC	JTI	14.9	2.1	3.0	0.0	−2.4	0.9	17.2	-3
		Premium	Dunhill Courtleigh	TTC	BAT	11.9	−0.1	1.0	−0.8	−11.4	−13	−0.4	−9.6
Madagascar	1.88	Value	Gauloises	TTC	Imperial Tobacco	N/A	N/A	N/A	34.9	N/A	N/A	N/A	34.9
		Popular	Parker and Simpson	TTC	Imperial Tobacco	N/A	N/A	N/A	10.1	N/A	N/A	N/A	10.1
		Premium	Lm	TTC	PMI	N/A	N/A	N/A	18.4	N/A	N/A	N/A	18.4
Malawi	N/A	Value	Brothers Menthol	Domestic	VITL	N/A	−0.4	−1.0	N/A	N/A	−21.3	−11.8	N/A
			Ascot Filter	TTC	BAT	N/A	1.6	2.5	−4.8	N/A	−5.8	10.8	−41
		Popular	Nyasa	Domestic	Nyasa	N/A	−22.8	7.6	−2.5	N/A	−79.5	69.8	−28.4
			Pall Mall Red	TTC	BAT	N/A	−4.7	13.7	−5.8	N/A	−32.6	56.5	−30.5
		Premium	Sino-ma	Domestic	Sino-Ma Lt.	N/A	0.6	−3.3	7.4	N/A	−12.6	−17.2	3.3
			Peter Stuyvesant Blue	TTC	BAT	N/A	−2.9	0.3	12.6	N/A	−20	−9.5	16.9
			Dunhill Master Blend	TTC	BAT	N/A	3.2	−1.7	7.3	N/A	−6.5	−14.1	4.4
Mozambique	2.50	Value	Caesar Blue	Domestic	BTC	N/A	N/A	N/A	2.1	N/A	N/A	N/A	−4.9
		Popular	Pall Mall Blue	TTC	BAT	N/A	N/A	N/A	0.8	N/A	N/A	N/A	−3.9
		Premium	Camel Classic	TTC	PMI	N/A	N/A	N/A	−5.0	N/A	N/A	N/A	−9.5
			Dunhill Double Capsule	TTC	BAT	N/A	N/A	N/A	−3.9	N/A	N/A	N/A	−7.8
Namibia	2.38	Value	LD Blue	TTC	JTI	2.9	0.4	2.1	1.0	32.2	−24.7	12.7	−20.8
		Popular	Chesterfield Blue	TTC	PMI	1.8	1.9	0.0	1.3	1.8	3.4	−12.3	−3.8
			Craven A Menthol	TTC	BAT	2.5	0.4	0.9	1.2	7.2	−7.2	−4.4	-4
			Camel Activate	TTC	JTI	1.3	0.3	−0.2	0.8	−2.3	−10.2	−16.5	−10.9
		Premium	Marlboro	TTC	PMI	1.9	0.9	0.7	2.2	2.3	−2.9	−4.5	3.6
			Kent	TTC	BAT	0.8	1.5	1.2	0.5	−3.7	0.4	−1.1	-7
Nigeria	1.25	Value	Oris	Domestic	N/A	N/A	N/A	33.0	12.6	N/A	N/A	−8.5	−26.1
			Winston Blue	TTC	JTI	N/A	N/A	47.1	22.8	N/A	N/A	5.4	−2.1
		Popular	London Menthol	TTC	BAT	N/A	N/A	32.1	13.0	N/A	N/A	-4	−18.3
			Dorchester St Moritz	TTC	JTI	N/A	N/A	25.8	13.9	N/A	N/A	−14.1	−19.2
		Premium	Dunhill Switch	TTC	BAT	N/A	N/A	113.7	−9.5	N/A	N/A	127.1	−35.9
South Africa	2.38	Value	Atlantic Menthol	Domestic	Carnilinx	1.5	2.4	2.7	1.0	−12.1	−43.8	80.7	−17.5
			Voyager Blue	Domestic	GLTC	1.1	2.8	1.2	0.7	−20	−45.6	64.5	−26.6
			Pall Mall Red	TTC	BAT	2.4	2.5	0.8	0.6	9.9	−17.3	−10.6	−16.8
		Popular	Chesterfield Blue	TTC	PMI	1.5	3.6	1.9	0.3	−1.7	−2.4	5.5	−9.8
			Craven A Menthol	TTC	BAT	2.7	2.9	1.3	0.8	8	−7.9	−0.1	−4.3
			Glamour Pinks	TTC	JTI	1.2	3.2	2.2	−0.1	−2.9	−5.5	6.8	−10.5
		Premium	Marlboro Gold	TTC	PMI	1.1	3.5	1.5	0.4	−3.3	−3.6	1.3	−6.1
			Dunhill Courtleigh	TTC	BAT	1.1	2.8	1.2	0.7	8.5	−7.6	−0.5	−4.2
			Camel Blue	TTC	JTI	0.7	3.8	1.7	0.1	−5.4	−1.7	3	−7.8
Tanzania	0.75	Value	Master	Domestic	Mastermind	N/A	3.8	14.9	−3.4	N/A	33.8	68.5	38.5
		Popular	Chesterfield Remix	TTC	PMI	N/A	1.3	5.7	N/A	N/A	12.5	22.3	43
			Club Menthol	TTC	JTI	N/A	N/A	N/A	10.7	N/A	29.3	22.2	38.5
		Premium	Embassy	Domestic	TCC	N/A	1.9	7.4	7.5	N/A	8.3	18.1	19.1
			Dunhill Blue	TTC	BAT	N/A	N/A	1.7	21.2	N/A	N/A	5.7	55.1
			Camel White	TTC	JTI	N/A	N/A	4.0	8.2	N/A	N/A	10.7	22
Zambia	1.38	Value	Guards Green	Domestic	RITCO	N/A	0.9	−2.6	N/A	N/A	−104.3	−13.5	N/A
			Safari Menthol	TTC	BAT	N/A	−6.4	−2.8	N/A	N/A	−118.5	−42.9	N/A
		Popular	Peter Stuyvesant Blue	TTC	BAT	N/A	−0.6	−4.7	N/A	N/A	−9.3	−12.9	N/A
		Premium	Camel Blue	TTC	JTI	N/A	N/A	−3.2	N/A	N/A	N/A	−5.8	N/A
			Dunhill Switch	TTC	BAT	N/A	−1.8	−4.4	N/A	N/A	−7.9	−8.1	N/A
Zimbabwe	1.25	Value	Remington Gold	Domestic	GLTC	1.5	−0.9	−44.0	−72.1	0	0	−46.8	−48
			Ascot Toasted	TTC	BAT	1.5	−0.9	−24.0	N/A	0	0	102.1	N/A
		Popular	Pacific Blue	Domestic	PCC	−3.6	11.1	−50.7	−74.4	−25.7	82.7	−73.7	−56
			Madison Toasted	TTC	BAT	1.5	20.8	−36.4	−83.6	1.1	94.5	−23.7	−91.9
		Premium	Branson Mint	Domestic	PCC	1.5	3.2	−55.1	−76.0	1.7	13.6	−81.3	−60
			Newbury Filter	TTC	BAT	4.7	0.1	−21.2	−81.6	12.9	2.3	29.9	−90.8

Brands were allocated to producers based on desktop research conducted by the authors; sources included company websites, legal documents and media articles, among others. Errors and omissions are possible.

Authors’ own calculation using the African Cigarette Price (ACP) Project and WHO’s Global Tobacco Epidemic Report.

Yellow: undershifting, Blue: overshifting, Pink: percentage change in tax more than 20% (arbitrarily chosen). (For colour information, please refer to the online version).

BAT, British American Tobacco; GLTC, Global Leaf Tobacco Company; IB, Imperial Brands; ITC, Independent Tobacco Company; JTI, Japan Tobacco International; NTE, National Tobacco Enterprise; PCC, Pacific Cigarette Company; PMI, Philip Morris International; RITCO, Roland Imperial Tobacco; TCC, Tanzania Cigarette Company; TTC, transnational tobacco company; VITL, Vision international Tobacco Limited.

#### Tax shifting between countries and over time

The only countries that show a predominant pattern of overshifting are Botswana and Tanzania (2018), even though their Tobacconomics Scores differ greatly (Botswana far outperforms Tanzania). In the case of Botswana, it was interesting to note that taxes were only undershifted in 2020 when there was a large increase in taxation.

Undershifting was more prevalent in Lesotho, SA, Zambia, and Malawi. Undershifting also occurred when tax changes were small, both when the tax increased and when it decreased in real terms (because it was not increased in nominal terms).

The case of Namibia highlights a lack of clear association with changes in taxation. In 2017, there was mainly overshifting of the small tax increases, but from 2018 this switched to undershifting, despite there being even smaller tax increases applied.

In 2020, a dominant strategy of undershifting is observed in most of the countries (except for Tanzania and Madagascar where overshifting was dominant) occurring both when taxes increased and when they did not.

#### Tax shifting by price segment

There is no clear evidence of value brands being undershifted more often (or to a greater degree) than popular or premium brands. Looking within country and year, tax shifting was similar across price segments. Exceptions to this include the value category in Namibia (2018 and 2019), Lesotho (2020), and Tanzania (2020). A mixed pattern emerged for Nigeria (overshifting taxes on premium and TTC’s value brands, others undershifted) and Malawi (overshifting popular and TTC’s value brands, others undershifted).

#### Tax shifting by producer

Domestic producers tended to sell only value products, with the exceptions of Malawi, Tanzania, and Zimbabwe, whose domestic producers also have brands in higher segments. Overall, the pricing strategies of domestic and TTC producers were similar, however, domestic producers tended to shift the tax to a greater degree (larger change in price). The pricing strategies of different TTCs within the same price segment were typically similar, with a few exceptions; PMI chose to overshift its premium brand in Namibia in 2017 and 2020 and undershift in 2018, while BAT was doing the opposite in those years. Similarly, BAT in SA was the only TTC to overshift taxes on all its brand segments in 2017, and the only one to undershift in 2019.

In Ethiopia, brands manufactured by National Tobacco Enterprise (NTE) or those that have a special license to be sold from NTE are considered legal while all others are illicit.^45^ The results show that between 2019 and 2020 all legal domestic and imported TTC brands were undershifted, while illicit products, including one brand from BAT, were overshifted despite the large increase in tax.

### Tax pass-through for single-sticks

There was less data available on single-sticks for most countries, so only a single brand is included in table 2 in each price segment (see also online supplemental appendix table 4 for nominal price information). Overall, taxes on single-sticks were typically undershifted for all price categories, brands, and companies. Exceptions include Madagascar, Tanzania, SA, and Zimbabwe. In all cases, there does not seem to be a clear association with the Tobacconomics Score or size of the tax change.

**Table 2:** Year-to-year percentage change in real net price (2017–2020), by brand category, producers (TTC or domestic) and companies for single-sticks

Countries	Tobacconomics tax scores	Brand segment	Brand name	Owned by TTC or domestic company	Tobacco company	Percentage change in real tax				Percentage change in net price			
						2017	2018	2019	2020	2017	2018	2019	2020
Botswana	4.13	Value	Peter Stuyvesant	Domestic	PCC	9.5	4.3	−17.3	43.8	−27.8	−6.8	4.5	−22.3
		Popular	Craven A Menthol	TTC	BAT	6.6	2.0	−26.7	60.3	−29.0	−6.3	12.1	−29.3
		Premium	Dunhill Menthol	TTC	BAT	6.2	3.4	−30.4	67.4	−24.4	−6.3	−13.4	−32.4
Ethiopia	1.5	Value	Nyala Filter	Domestic	NTE	N/A	N/A	46.1	96.9	N/A	N/A	−25.6	−63.8
		Popular	Rothmans Blue	TTC	BAT	N/A	N/A	16.9	35.5	N/A	N/A	−18.8	−30.0
		Premium	Marlboro Red	TTC	PMI	N/A	N/A	12.0	−19.7	N/A	N/A	−17.7	−30.7
Lesotho	2.38	Value	Sun White	Domestic	Sun Tobacco	13.2	−6.0	5.2	2.5	−83.1	6.7	244.1	−17.1
		Popular	Peter Stuyvesant Filter	TTC	BAT	15.0	−1.3	1.7	−0.3	−16.1	−6.1	23.1	−7.5
		Premium	Dunhill Courtleigh	TTC	BAT	11.9	−0.1	1.0	−0.8	−13.1	−6.2	−7.5	−7.3
Madagascar	1.88	Value	New Red	TTC	IB	N/A	N/A	N/A	34.9	N/A	N/A	N/A	71.2
		Popular	MÃLia	TTC	IB	N/A	N/A	N/A	10.1	N/A	N/A	N/A	−95.0
		Premium	Good Look	TTC	PMI	N/A	N/A	N/A	18.4	N/A	N/A	N/A	18.4
Mozambique	2.5	Value	Caesar Blue	Domestic	BTC	N/A	N/A	N/A	2.1	N/A	N/A	N/A	−60.2
		Popular	Pall Mall Blue	TTC	BAT	N/A	N/A	N/A	0.8	N/A	N/A	N/A	−3.6
		Premium	Dunhill Double Capsule	TTC	BAT	N/A	N/A	N/A	−3.9	N/A	N/A	N/A	−2.5
Namibia	2.38	Value	Aspen	TTC	JTI	2.9	0.4	2.1	N/A	−12.3	−52.2	403.4	N/A
		Popular	Pall Mall Blue	TTC	PMI	2.5	0.4	0.9	N/A	−13.3	−11.5	40.0	N/A
		Premium	Dunhill Kingsize	TTC	PMI	0.8	1.5	1.2	N/A	−9.4	−9.3	42.6	N/A
Nigeria	1.25	Value	Oris	Domestic	N/A	N/A	N/A	33.0	12.6	N/A	N/A	−27.4	−26.1
		Popular	Dorchester St Moritz	TTC	JTI	N/A	N/A	25.8	13.9	N/A	N/A	101.6	−45.5
		Premium	Dunhill Switch	TTC	BAT	N/A	N/A	113.7	−9.5	N/A	N/A	191.4	9.3
South Africa	2.38	Value	Rg Blue	Domestic	Carnilinx	1.5	2.4	2.7	1.0	−244.7	−126.0	439.1	78.0
		Popular	Pall Mall Blue	TTC	PMI	2.4	2.5	0.8	0.6	−51.3	−15.5	160.6	−38.9
		Premium	Dunhill Courtleigh	TTC	BAT	1.1	2.8	1.2	0.7	19.0	−8.6	17.2	−6.1
Tanzania	0.75	Value	Master Filter	Domestic	Mastermind	N/A	3.8	14.9	−3.4	N/A	68.4	−11.0	46.1
		Popular	Club Filter	TTC	JTI	N/A	1.3	9.5	10.7	N/A	60.3	−9.1	38.5
		Premium	Embassy	Domestic	TCC	N/A	1.9	7.4	7.5	N/A	25.3	−6.9	19.1
Zambia	1.38	Value	Guards Green	Domestic	RITCO	N/A	N/A	0.9	N/A	N/A	N/A	42.1	N/A
		Popular	Life Menthol	TTC	BAT	N/A	N/A	−0.7	N/A	N/A	N/A	−198.8	N/A
		Premium	Peter Stuyvesant	TTC	JTI	N/A	N/A	−1.8	N/A	N/A	N/A	−96.7	N/A
Zimbabwe	1.25	Value	Pacific Blue	Domestic	GLTC	−3.6	11.1	−50.7	N/A	8.2	−13.2	277.8	N/A
		Popular	Madison Toasted	TTC	BAT	1.5	20.8	−36.4	N/A	1.5	−10.1	83.5	N/A
		Premium	Kingsgate	Domestic	PCC	4.7	0.1	−21.2	N/A	0.1	−1.4	129.9	N/A

Brands were allocated to producers based on desktop research conducted by the authors; sources included company websites, legal documents and media articles, among others. Therefore, errors and omissions are possible.

Authors’ own calculation using the database of African Cigarette Price (ACP) Project and WHO’s Global Tobacco Epidemic Report.

Yellow: undershifting, Blue: overshifting, Pink: percentage change in tax more than 20% (arbitrarily chosen). (For colour information, please refer to the online version).

Since there were no data on single-sticks from Malawi, therefore it is not added to the table.

BAT, British American Tobacco; GLTC, Global Leaf Tobacco Company; IB, Imperial Brands; ITC, Independent Tobacco Company; JTI, Japan Tobacco International; NTE, National Tobacco Enterprise; PCC, Pacific Cigarette Company; PMI, Philip Morris International; RITCO, Roland Imperial Tobacco; TCC, Tanzania Cigarette Company; TTC, transnational tobacco company; VITL, Vision international Tobacco Limited.

### Summary results

Table 3 provides a summary of the of tax pass-through results. It can be observed that the strategy of undershifting was more readily used by tobacco companies in SSA and more so in 2020. Overall, single-stick pass-through followed a similar trend to packs. Exceptions to this are Botswana, Madagascar, Tanzania, and Zimbabwe, which had more occurrences of overshifting with single-sticks than other countries.

**Table 3:** Summary table of tax pass-through strategies

Countries	Presentation	Data notes	Shifting of taxes			
			2017	2018	2019	2020
Botswana	Pack of 20	Data available for all the years. 13 brands	Overshifting	Overshifting (except Rothmans)	Overshifting	Undershifting
	Single	Data available for all the years. 4 brands	Undershifting	Undershifting	Overshifting (except undershifting for premium brands)	Undershifting
Ethiopia	Pack of 20	Data available for 2019 and 2020. 8 brands	N/A	N/A	N/A	Undershifting (except overshifting for illicit brands)
	Single	Data available for 2018 to 2020. 8 brands	N/A	N/A	Undershifting (except overshifting for an illicit brand)	Undershifting
Lesotho	Pack of 20	Data available for all the years. 7 brands	Undershifting (except overshifting for Peter Stuyvesant)	Undershifting (except overshifting for Rothmans and Camel)	Overshifting (except undershifting for BAT’s International brands)	Undershifting (except illicit cigarette—Sun overshifting)
	Single	Data available for all the years. 15 brands	Undershifting (except Kent overshifting)	Mix pattern (7 value and popular brands overshifted, 5 premium brands undershifted)	Mix pattern (7 brands overshifted, 8 undershifted)	Mix pattern (5 brands overshifted, 8 undershifted)
Madagascar	Pack of 20	Data available for 2019 and 2020. 8 brands	N/A	N/A	N/A	Overshifting
	Single	Data available for 2019 and 2020. 7 brands	N/A	N/A	N/A	Overshifting (except undershifting for 2 illicit brands)
Malawi	Pack of 20	Data available for 2017 to 2020. 15 brands	N/A	Undershifting	Mix pattern (7 brands overshifted, 8 undershifted)	Mix Pattern (7 brands overshifted, 5 undershifted)
Mozambique	Pack of 20	Data available for 2019 and 2020. 7 brands	N/A	N/A	N/A	Undershifting
	Single	Data available for 2019 and 2020. 7 brands	N/A	N/A	N/A	Undershifting (except overshifting for Ld)
Namibia	Pack of 20	Data available for all the years. 15 brands	Mix Pattern (9 brands overshifted, 6 undershifted)	Undershifting (except overshifting for Aspen, Chesterfield, Dunhill, Kent and Vogue)	Undershifting (except overshifting for Aspen and LD)	Undershifting (except overshifting for Marlboro)
	Single	Data available for 2016–2019. 8 brands	Undershifting (except overshifting for an illicit cigarette—yes)	Undershifting (except overshifting for an illicit cigarette—yes)	Overshifting (except undershifting for an illicit cigarette—yes)	N/A
Nigeria	Pack of 20	Data available for 2019 and 2020. 13 brands	N/A	N/A	Mix Pattern (5 brands overshifted, 6 undershifted)	Undershifting
	Single	Data available for 2018, 2019 and 2020. 10 brands	N/A	N/A	Overshifting (except undershifting for Pall Mall)	Undershifting (except overshifting for Dunhill)
South Africa	Pack of 20	Data available for all the years. 33 brands	Undershifting (except 4 BAT brands overshifted: Benson & Hedges, Craven A, Pall Mall and Peter Stuyvesant)	Undershifting (except a domestic brand overshifting)	Overshifting (except undershifting for Dunhill, Embassy, Pacific, Pall Mall, Rothmans and Vogue)	Undershifting (except overshifting for Domestic brands)
	Single	Data available for all the years. 16 brands	Undershifting (except overshifting for premium brands)	Undershifting (except overshifting for Craven A, RG and Rothmans)	Overshifting (except undershifting for Benson & Hedges, Craven A, Peter Stuyvesant and Rothmans)	Undershifting (except overshifting for Benson & Hedges, Craven A, Kent, RG and Rothmans)
Tanzania	Pack of 20	Data available for 2017 to 2020. 12 brands	N/A	Overshifting	Overshifting	Overshifting
	Single	Data available for 2017 to 2020. 12 brands	N/A	Overshifting	Undershifting (except overshifting for Marlboro and Sm)	Overshifting
Zambia	Pack of 20	Data available for 2017 to 2019. 1 brand	N/A	Undershifting (except Sweet Menthol)	Undershifting	N/A
	Single	Data available for 2018 and 2019. 6 brands	N/A	N/A	Undershifting	N/A
Zimbabwe	Pack of 20	Data available for all the years. 17 brands	Overshifting (except undershifting for Pacific)	Overshifting (except linear shifting for domestic brands: Mega and Remington)	Mix pattern (domestic brands undershifted and TTCs overshifted)	Undershifting
	Single	Data available for 2016–2019. 14 brands	Overshifting (data only available for 3 brands)	Undershifting (except overshifting for Chelsea and Dunhill)	Overshifting (except undershifting for Dunhill, Newbury and Roxbury)	N/A

Authors’ own calculation using the database of African Cigarette Price (ACP) Project and WHO’s Global Tobacco Epidemic Report.

Yellow: undershifting, Blue: overshifting. (For colour information, please refer to the online version).

* Since there were no data on single-sticks from Malawi, therefore it is not added to the table.

## Discussion

The findings of this study reveal that taxes on cigarettes are not perfectly passed on to smokers in the SSA countries and hence the effectiveness of tobacco tax changes is impacted by the TI’s pricing strategies. We found evidence that both domestic and TTCs differentially shifted taxes. These findings are in line with previous studies from many HICs^22 29 30 46 47^ and LMICs^2748^,^51^ for cigarette packs.^52^ However, the tax pass-through on single-sticks were in contrast to the only previous study on singles from Colombia,^32^ which showed a pattern of overshifting as opposed to the undershifting observed in this study.

The observed industry pricing strategies differed in each market, over time and by tax level/type, plausibly because of changes in market dynamics and trading environment, and with the final chosen price likely determined by acceptable nominal values. A fitting overall illustration is Mozambique, which ratified the FCTC in 2017 and had the largest increase in Tobacconomics Score in 2020, due to a change from a tiered to a uniform-specific tax structure, which may be the cause of the undershifting observed in recent years.^53^ Similarly, in Nigeria TTCs undershifted taxes after the introduction of a specific tax element in 2018–2019.^54 55^ Moreover, in Madagascar—which is one of only three countries across the continent (along with Mauritius and Egypt)^56^ with excise taxes in line with FCTC guidelines—the TTC (Imperial brands) overshifted taxes. This is consistent with the fact that overshifting has been documented in Mauritius^43^ and HICs with relatively high excise taxes such as the UK,^15 57^ the USA,^2958^,^60^ Ireland,^23^ New Zealand^22^ and Taiwan.^61^ In east Africa, JTI overshifted taxes in Tanzania which is a major market compared with Ethiopia where JTI made an acquisition of the NTE (in 2017) and chose to absorb taxes, perhaps as it considered this an emerging market with fast track growth in consumption.^62^ There is evidence to suggest that the percentage of smokers is increasing, especially among females, and tax undershifting may be a factor in this.^63^

Four of the included countries (Botswana, Lesotho, Namibia, and SA) are members of the Southern African Customs Union (SACU), and thus apply the same specific excise taxes and import tariffs, as determined by SA (SACU Agreement, 1969). These countries have a relatively high excise tax proportion of price among SSA countries (although the Tobacconomics Score is fairly low), and here the TTCs were found to predominantly undershift taxes on their cigarettes. The exception was Botswana, where the government imposes an additional levy on tobacco products, giving it the highest tobacco taxes and prices in SACU, including a greater than 20% increase in real taxes in 2020^64 65^ (and also the lowest smoking prevalence of the group).^66^ We see a very different trend to that in SA, Lesotho, and Namibia, where almost exclusive undershifting for packs was seen between 2017 and 2019.

For single-sticks, it is possible that sales occur at rigid (nominal) currency price points (eg, ZAR1, ZAR1.5 ZAR2 in SA), which make incremental price changes hard to apply (online supplemental appendix table 4). It might also be these sales are non-duty-paid illicit and hence not directly impacted by tax changes. Single-sticks are still being sold in SSA despite laws prohibiting their sales in some countries,^67^ and their per-unit-price is also lower than for packs in some markets such as Lesotho and Ethiopia.^34^ Empirical studies identify TTCs as the main perpetrators of the single-stick sales^68^ on the continent by encouraging informal channels to supply markets and mobilising them to lobby against regulations.^32 33 40 69^ This suggests weak enforcement of regulating and controlling the distribution of cigarettes. Given that single-stick sales undermine tobacco control policies, there is a crucial need for strengthened regulations, including greater enforcement and/or penalties, to prohibit such sales in SSA countries.

The frequent use of overshifting suggests that there remains scope for further tax increases in SSA. The TI has a reputation for aggressively opposing tobacco taxation, often arguing that it leads to illicit trade. However, these allegations are inconsistent with their observed pricing behaviours. If they were truly worried about higher prices driving the illicit market, they would not overshift taxes.^15^ Instead, the TI pushes for low excise duties leaving room to increase its prices and maximise its profits; governments could instead be taking this revenue as taxation rather than allowing the TI to make larger profits.^70 71^ Empirical evidence points to non-price factors such as issues surrounding tax administration, in particular a lack of tax enforcement, as important determinants of illicit trade.^72^,^74^ Additionally, evidence suggests even in the presence of an illicit market governments continue to raise revenues by increasing taxes^75 76^ that can then help fund better enforcement mechanisms such as an independent track and trace system.^77^

In addition, undershifting behaviour reveals that the TI has profit margins that are high enough for them to reduce net price while still making a profit. Hence, the presence of undershifting also illustrates that there is scope for governments to increase taxes. Indeed, whether the TI chooses to undershift, overshift or perfectly shift a tax increase will also be a function of their strategic goals in that country, at that time, whether to maximise profit per consumer (increase margins), or reduce margins to increase the consumer base.

### Strengths and Limitations

To the best of our knowledge, this is the first comprehensive multi-country academic study from SSA that generates empirical findings on TI’s price responses to tax increases for cigarette packs and single-sticks. Also, the first study that examines the pricing behaviours of tobacco companies in detail, especially in LMICs. The findings of the study expand the evidence base on TI pricing strategies in response to excise taxes especially in the under-researched African context.

Because GTR only reports information on taxes in 2 yearly intervals, we use linear interpolation for the omitted years, which could have created errors. However, we verified the data with alternative sources, and no major inconsistencies were identified. Another limitation of the GTR data was that the within-year timing of it was unclear, so we could not assess when tax changes were introduced/applied and hence the extent to which information for a given year aligned with the ACP data. The limitations related to the non-representative nature of the ACP project data have been described previously^34^ but one that is inherent to this study is related to the selection of popular brands in each price segment for analysis, where the choices may not be true representatives of the markets. A key limitation of using the ACP data in our analysis is that for some countries the datasets had information for only 2 or 3 years, for example, Mozambique, Madagascar, and Ethiopia, thereby negating exploration of long-term trends. Furthermore, 2020 ACP data was gathered in January, rather than mid-year (due to the COVID-19 pandemic), which could have created bias if price changes were generally applied mid-year. Finally, the nature of the ACP data required us to assume all data points were duty-paid, but some could have been non-duty-paid. Such sales might have created some bias in our findings, although our use of median (rather than mean) values should minimise such bias. Given the nature of our data, we could also not explore any of the other price responses of the TI to taxation (eg, price smoothing), so we cannot conclude whether the strategies not examined are present in the market or not. However, considering the lack of comprehensive and reliable country level data on pricing and taxation, and the scarcity of research on this topic in SSA, the WHO GTR database ensured a level of reliability and consistency, and hence the findings of this study are pertinent.

### Policy recommendation

Governments and policymakers in SSA countries must regularly monitor how the industry responds to tax changes and then react appropriately to ensure that excise tax increases are effectively increasing price. Measures to do this include limiting the number of official price changes possible in the market, implementing larger and unannounced specific excise taxes, along with the adoption of minimum excise tax laws (where ad valorem duty applies). Governments should be mindful that different price segments give the industry flexibility in how they respond to higher taxes. Given we have found variability in this regard, such uncertainly about industry actions could make it challenging to accurately predict changes to tax revenue and prevalence. Our findings also reinforce the importance of banning single-sticks and better enforcement of laws doing so, as their availability hinders the impact of many types of tobacco control policy, including tax.

## Conclusion

This paper provides a description of TI’s pricing strategies in response to tax increases in 12 SSA countries. It also provides recommendations which are applicable, and beneficial, for policy makers and advocates in all countries with similar income levels for the effective implementation of targeted local tax policies. It also highlights the need for improved data within the region, particularly to buttress these findings for SSA countries not included in our study and to monitor the effectiveness of our recommended policy measures.

## Supplementary material

10.1136/tc-2023-058054 online supplemental table 1

## Data Availability

Data are available in a public, open access repository.
