# Supplementary material for: How has the tobacco industry passed tax changes through to consumers in 12 sub-Saharan African countries?
Source: Tob Control. 2023 Aug 11;34(1):e058054. doi: 10.1136/tc-2023-058054 (PMC11877087; doi:10.1136/tc-2023-058054)
Supplement: online supplemental table 1 [file tc-34-1-s001.pdf]

Appendices

Cigarette smoking prevalence across the region is the highest in Lesotho (18%) and SA (17%), and lowest are in Ethiopia and Nigeria (3%). The overall Tobacconomics tax score is highest for Botswana (4.13) and lowest for Ghana and Tanzania (0.75). Madagascar has the highest total and excise (80.4% & 63.6%) tax proportion of the retail prices.

Appendix Table I: Background characteristics of included SSA countries (2020)

**Countries**	**Income level***	**Population**	**Smoking Prevalence**	**Tax Structure**	**Total taxes on retail prices**	**Excise tax proportion of price**	**Tax Scores**	**Price of a pack of 20 of the most sold brand (in US$)**	**Market Lead****
Botswana	UMI	2.35 M	13%	Specific	52.2%	35.15%	4.13	4.31	BAT
Ethiopia	LI	115 M	3%	Mixed	51.2%	40.78%	1.50	1.15	JTI
Lesotho	LMI	2.14 M	18%	Specific	50.6%	37.54%	2.38	2.77	BAT
Madagascar	LI	27.69 M	13%	Ad valorem	80.4%	63.61%	1.88	1.05	Imperial
Malawi	LI	19.31 M	7%	Specific	56.3%	42.16%	--	0.71	JTI
Mozambique	LI	31.26 M	11%	Specific	28.5%	14.00%	2.50	0.85	BAT
Namibia	UMI	2.54 M	14%	Specific	42.0%	29.00%	2.38	3.58	BAT
Nigeria	LMI	206.1 M	3%	Mixed	35.1%	30.00%	1.25	1.05	BAT
South Africa	UMI	59.31 M	17%	Specific	52.7%	39.69%	2.38	2.62	BAT
Tanzania	LMI	59.73 M	6%	Specific	30.0%	14.71%	0.75	1.74	JTI
Zambia	LI	18.38 M	10%	Specific	38.8%	25.00%	1.38	1.16	BAT

Zimbabwe	LMI	14.86 M	8%	Mixed	29.3%	16.25%	1.25	1.24	BAT

+Data sources For Population: World Bank Statistics, For Tax scores: Tobacconomics Cigarette Tax Scorecard 2nd edition, For smoking prevalence, prices, and tax share: GTR 2021
*UMI: Upper middle income, LMI: Lower middle income and LI: Low income
**BAT: British American Tobacco, JTI: Japan Tobacco International

Appendix Table II: Currencies, Acronyms, and exchange rates

Country	Acronym	Full currency name	National currency per US dollar (2018)
Botswana	BWP	Botswana Pula	10.2
Ethiopia	ETB	Ethiopian Birr	27.4515
Lesotho	LSL	Lesotho Loti	11.8376
Madagascar	MGA	Malagasy Aviary	3334.75
Malawi	MWK	Malawian Kwacha	726.7629
Mozambique	MZN	Mozambican Metical	60.33
Namibia	NAD	Namibian Dollar	11.8533
Nigeria	NGN	Nigerian Naira	359.9049
South Africa	ZAR	South African Rand	13.234
Tanzania	TZS	Tanzanian Shilling	2250.2346
Zambia	ZMW	Zambian Kwacha	10.458
Zimbabwe	USD	United states Dollar	1
Exchange rates from the Organisation for Economic Co-operation and Development for January 2018 Website: https://data.oecd.org/conversion/exchange-rates.htm			

Appendix Table III: Total (Real) Tax information for packs of 20 cigarettes for all the countries all the years

Countries (Currency)	Brand Name	N	Price and Tax 2016				N	Price and Tax 2017				N	Price and Tax 2018				N	Price and Tax 2019				N	Price and Tax 2020			
			local price	specific tax	Other taxes	total tax		local price	specific tax	Other taxes	total tax		local price	specific tax	Other taxes	total tax		local price	specific tax	Other taxes	total tax		local price	specific tax	Other taxes	total tax
Botswana (BWP)	Pacific Blue	10	23.52	10.49	4.72	15.21	3	26.34	11.36	5.29	16.65	69	27.95	12.13	5.23	17.37	50	27.08	14.31	0.05	14.36	52	24.98	16.4	4.25	20.65
	Chesterfield Blue	12	36.64	10.49	7.35	17.84	5	37.14	11.36	7.45	18.82	32	38.70	12.13	7.24	19.38	101	39.24	14.31	0.07	14.39	41	38.60	16.40	6.57	22.97
	Craven A Menthol	11	37.65	10.49	7.56	18.05	19	39.20	11.36	7.87	19.23	81	39.95	12.13	7.48	19.61	220	37.74	14.31	0.07	14.38	96	39.12	16.40	6.66	23.06
	Rothmans Blue	5	39.24	10.49	7.88	18.37	9	40.55	11.36	8.14	19.50	21	39.65	12.13	7.42	19.56	11	39.49	14.31	0.07	14.39	8	38.32	16.40	6.52	22.92
	Marlboro Blue Ice	8	39.24	10.49	7.88	18.37	7	44.37	11.36	8.90	20.27	33	45.95	12.13	8.60	20.73	69	45.49	14.31	0.09	14.40	41	47.74	16.40	8.13	24.53
	Dunhill Menthol	13	41.68	10.49	8.37	18.86	16	43.13	11.36	8.66	20.02	12	45.75	12.13	8.56	20.70	19	44.53	14.31	0.08	14.40	16	45.20	16.40	7.69	24.09
	Camel Classic	4	49.86	10.49	10.01	20.50	6	51.60	11.36	10.36	21.72	24	53.95	12.13	10.10	22.23	31	53.25	14.31	0.10	14.41	28	51.80	16.40	8.82	25.22
	Total number of observations	75					94					316					570					333				

Ethiopia (ETB)	Nyala Filter	N/A	N/A	N/A	N/A	N/A	N/A	N/A	N/A	N/A	N/A	N/A	N/A	N/A	N/A	N/A	108	21.97	0.00	5.48	5.48	7	15.18	6.07	4.73	10.80
	Business Royals	N/A	N/A	N/A	N/A	N/A	N/A	N/A	N/A	N/A	N/A	N/A	N/A	N/A	N/A	N/A	69	17.57	0.00	4.39	4.39	4	30.35	6.07	9.46	15.53
	Benson & Hedges Filter	N/A	N/A	N/A	N/A	N/A	N/A	N/A	N/A	N/A	N/A	N/A	N/A	N/A	N/A	N/A	55	37.35	0.00	9.32	9.32	5	60.71	6.07	18.92	24.99
	Winston Blue	N/A	N/A	N/A	N/A	N/A	N/A	N/A	N/A	N/A	N/A	N/A	N/A	N/A	N/A	N/A	12	43.94	0.00	10.97	10.97	3	45.53	6.07	14.19	20.26
	Rothmans Blue	N/A	N/A	N/A	N/A	N/A	N/A	N/A	N/A	N/A	N/A	N/A	N/A	N/A	N/A	N/A	528	87.87	0.00	21.94	21.94	26	75.88	6.07	23.65	29.72
	Marlboro Red	N/A	N/A	N/A	N/A	N/A	N/A	N/A	N/A	N/A	N/A	N/A	N/A	N/A	N/A	N/A	20	101.05	0.00	25.23	25.23	4	45.53	6.07	14.19	20.26
	Total number of observations	N/A					N/A					N/A					804					58				
Lesotho (LSL)	Sun White	14	22.26	12.91	2.90	15.81	33	20.88	15.18	2.72	17.90	2	10.00	15.52	1.30	16.82	33	15.27	15.71	1.99	17.70	11	18.14	15.78	2.37	18.15
	Peter Stuyvesant Filter	22	36.73	12.91	4.79	17.70	62	39.67	15.18	5.17	20.35	4	35.00	15.52	4.57	20.09	117	36.25	15.71	4.73	20.43	30	35.15	15.78	4.58	20.37
	Camel Activate	22	32.26	12.91	4.21	17.12	79	34.45	15.18	4.49	19.67	2	35.00	15.52	4.57	20.09	55	38.17	15.71	4.98	20.68	18	37.64	15.78	4.91	20.69
	Dunhill Courtleigh	17	42.29	12.91	5.52	18.43	107	41.76	15.18	5.45	20.63	4	39.00	15.52	5.09	20.61	153	39.12	15.71	5.10	20.81	46	37.19	15.78	4.85	20.63
	Total number of observations	81					314					18					438					125				
Madagascar (MGA)	Gauloises	N/A	N/A	N/A	N/A	N/A	N/A	N/A	N/A	N/A	N/A	N/A	N/A	N/A	N/A	N/A	18	1841.62	0.00	1481.17	1481.17	3	2485.14	0.00	1998.74	1998.74
	Parker and Simpson	N/A	N/A	N/A	N/A	N/A	N/A	N/A	N/A	N/A	N/A	N/A	N/A	N/A	N/A	N/A	34	3950.28	0.00	3177.11	3177.11	5	4351.18	0.00	3499.54	3499.54
	Lm	N/A	N/A	N/A	N/A	N/A	N/A	N/A	N/A	N/A	N/A	N/A	N/A	N/A	N/A	N/A	27	5524.86	0.00	4443.51	4443.51	5	6539.85	0.00	5259.83	5259.83
	Total number of observations	N/A					N/A					N/A					121					25				

Malawi (MWK)	Brothers Menthol	N/A	N/A	N/A	N/A	N/A	10	446.00	189.36	45.27	234.63	19	400.00	187.70	45.96	233.66	93	378.11	182.88	48.47	231.36	4	N/A	N/A	N/A	N/A
	Ascot Filter	N/A	N/A	N/A	N/A	N/A	11	511.79	189.36	51.95	241.31	7	500.00	187.70	57.45	245.15	27	533.81	182.88	68.43	251.32	3	405.80	181.69	57.46	239.16
	Nyasa	N/A	N/A	N/A	N/A	N/A	7	1115.00	189.36	113.17	302.53	14	400.00	187.70	45.96	233.66	86	533.81	182.88	68.43	251.32	2	447.28	181.69	63.33	245.03
	Pall Mall Red	N/A	N/A	N/A	N/A	N/A	9	836.25	189.36	84.88	274.24	5	640.00	187.70	73.54	261.24	85	889.68	182.88	114.06	296.94	3	691.25	181.69	97.88	279.57
	Sino-ma	N/A	N/A	N/A	N/A	N/A	4	1887.42	189.36	191.57	380.93	33	1700.00	187.70	195.33	383.03	230	1461.30	182.88	187.34	370.22	4	1524.82	181.69	215.91	397.61
	Peter Stuyvesant Blue	N/A	N/A	N/A	N/A	N/A	13	1600.03	189.36	162.40	351.76	22	1340.00	187.70	153.97	341.67	217	1245.55	182.88	159.68	342.56	5	1441.46	181.69	204.11	385.80
	Dunhill Master Blend	N/A	N/A	N/A	N/A	N/A	5	1494.09	189.36	151.65	341.01	10	1430.00	187.70	164.31	352.01	54	1272.24	182.88	163.10	345.98	2	1337.77	181.69	189.43	371.12
	Total number of observations	N/A					58					159					911					24				
Mozambique (MZN)	Caesar Blue	N/A	N/A	N/A	N/A	N/A	N/A	N/A	N/A	N/A	N/A	N/A	N/A	N/A	N/A	N/A	6	43.31	7.41	6.29	13.70	8	42.13	7.86	6.12	13.99
	Pall Mall Blue	N/A	N/A	N/A	N/A	N/A	N/A	N/A	N/A	N/A	N/A	N/A	N/A	N/A	N/A	N/A	4	77.00	7.41	11.19	18.60	13	74.90	7.86	10.88	18.75
	Camel Classic	N/A	N/A	N/A	N/A	N/A	N/A	N/A	N/A	N/A	N/A	N/A	N/A	N/A	N/A	N/A	24	158.81	7.41	23.07	30.49	20	145.12	7.86	21.09	28.95
	Dunhill Double Capsule	N/A	N/A	N/A	N/A	N/A	N/A	N/A	N/A	N/A	N/A	N/A	N/A	N/A	N/A	N/A	6	161.21	7.41	23.42	30.84	6	149.80	7.86	21.77	29.63
	Total number of observations	N/A					N/A					N/A					45					54				
Namibia (NAD)	LD Blue	46	22.42	14.99	2.92	17.91	59	24.39	15.26	3.18	18.44	182	23.00	15.52	3.00	18.52	135	23.96	15.78	3.13	18.91	17	23.10	16.09	3.01	19.10
	Chesterfield Blue	36	35.42	14.99	4.62	19.61	55	36.06	15.26	4.70	19.96	84	36.99	15.52	4.82	20.34	55	34.95	15.78	4.56	20.34	16	34.66	16.09	4.52	20.61
	Craven A Menthol	105	38.48	14.99	5.02	20.01	187	40.32	15.26	5.26	20.52	209	38.99	15.52	5.09	20.61	187	38.35	15.78	5.00	20.78	16	37.90	16.09	4.94	21.03
	Camel Activate	88	35.65	14.99	4.65	19.64	160	35.53	15.26	4.63	19.89	151	33.99	15.52	4.43	19.95	160	31.63	15.78	4.13	19.91	19	30.50	16.09	3.98	20.07
	Marlboro	35	43.57	14.99	5.68	20.67	62	44.48	15.26	5.80	21.06	83	43.99	15.52	5.74	21.26	62	43.12	15.78	5.62	21.41	11	44.37	16.09	5.79	21.87
	Kent	108	45.27	14.99	5.91	20.89	197	44.55	15.26	5.81	21.07	176	44.97	15.52	5.87	21.39	197	44.97	15.78	5.87	21.65	10	43.45	16.09	5.67	21.75
	Total number of observations	1010					1881					2058					1230					216				

Nigeria (NGN)	Oris	N/A	N/A	N/A	N/A	N/A	N/A	N/A	N/A	N/A	N/A	5	170.00	20.00	35.08	55.08	9	178.41	34.79	38.46	73.25	10	160.15	46.44	36.00	82.45
	Winston Blue	N/A	N/A	N/A	N/A	N/A	N/A	N/A	N/A	N/A	N/A	4	100.00	20.00	20.63	40.63	6	267.61	34.79	57.69	92.48	7	120.12	46.44	27.00	73.45
	London Menthol	N/A	N/A	N/A	N/A	N/A	N/A	N/A	N/A	N/A	N/A	3	200.00	20.00	41.27	61.27	4	214.09	34.79	46.15	80.94	9	200.19	46.44	45.00	91.45
	Dorchester St Moritz	N/A	N/A	N/A	N/A	N/A	N/A	N/A	N/A	N/A	N/A	5	200.00	20.00	41.27	61.27	8	196.24	34.79	42.31	77.10	11	184.18	46.44	41.40	87.85
	Dunhill Switch	N/A	N/A	N/A	N/A	N/A	N/A	N/A	N/A	N/A	N/A	4	200.00	20.00	41.27	61.27	7	446.02	34.79	96.15	130.94	5	320.31	46.44	72.01	118.45
	Total number of observations	N/A					N/A					33					71					75				
South Africa (ZAR)	Atlantic Menthol	147	21.25	14.85	2.61	17.46	83	21.04	15.13	2.58	17.71	212	20.00	15.52	2.61	18.13	133	22.00	15.75	2.87	18.62	95	21.60	15.99	2.82	18.81
	Voyager Bleue	53	42.60	14.85	5.23	20.08	26	42.08	15.13	5.17	20.30	98	41.00	15.52	5.35	20.87	156	41.15	15.75	5.37	21.12	105	40.45	15.99	5.27	21.27
	Pall Mall Red	58	26.35	14.85	3.24	18.08	52	27.60	15.13	3.39	18.52	312	26.50	15.52	3.46	18.98	116	25.84	15.75	3.37	19.12	57	24.82	15.99	3.24	19.23
	Chesterfield Blue	23	33.63	14.85	4.13	18.98	83	33.66	15.13	4.13	19.26	49	34.00	15.52	4.43	19.95	106	35.16	15.75	4.59	20.34	9	33.77	15.99	4.40	20.40
	Craven A Menthol	128	37.01	14.85	4.54	19.39	108	38.92	15.13	4.78	19.91	179	38.00	15.52	4.96	20.48	437	38.28	15.75	4.99	20.74	42	37.69	15.99	4.91	20.91
	Glamour Pinks	36	39.24	14.85	4.82	19.67	38	38.91	15.13	4.78	19.91	62	38.50	15.52	5.02	20.54	261	40.18	15.75	5.24	20.99	222	38.15	15.99	4.97	20.97
	Marlboro Gold	56	42.60	14.85	5.23	20.08	149	42.08	15.13	5.17	20.30	74	42.00	15.52	5.48	21.00	67	42.58	15.75	5.55	21.31	44	41.37	15.99	5.39	21.39
	Dunhill Courtleigh	136	42.61	14.85	5.23	20.08	79	42.08	15.13	5.17	20.30	212	41.00	15.52	5.35	20.87	96	41.15	15.75	5.37	21.12	10	40.45	15.99	5.27	21.27
	Camel Blue	39	43.74	14.85	5.37	20.22	65	42.61	15.13	5.23	20.36	54	43.00	15.52	5.61	21.13	94	44.02	15.75	5.74	21.49	39	42.29	15.99	5.51	21.51
	Total number of observations	807					801					1609					2219					1040				

Tanzania (TZS)	Master	N/A	N/A	N/A	N/A	N/A	3	1684.80	590.19	257.00	847.19	9	2000.00	574.49	305.08	879.57	24	2898.55	568.60	442.15	1010.75	12	2800.53	549.37	427.20	976.57
	Chesterfield Remix	N/A	N/A	N/A	N/A	N/A	2	2316.60	590.19	353.38	943.57	6	2500.00	574.49	381.36	955.85	39	2898.55	568.60	442.15	1010.75	62	N/A	N/A	N/A	N/A
	Club Menthol	N/A	N/A	N/A	N/A	N/A	3	2106.00	590.19	321.25	911.44	13	N/A	N/A	N/A	N/A	62	2898.55	568.60	442.15	1010.75	32	3734.04	549.37	569.60	1118.97
	Embassy	N/A	N/A	N/A	N/A	N/A	3	3948.75	590.19	602.35	1192.54	7	4200.00	574.49	640.68	1215.17	10	4830.92	568.60	736.92	1305.52	31	5601.06	549.37	854.40	1403.77
	Dunhill Blue	N/A	N/A	N/A	N/A	N/A	2	N/A	N/A	N/A	N/A	5	3700.00	574.49	564.41	1138.90	74	3864.73	568.60	589.54	1158.13	52	5601.06	549.37	854.40	1403.77
	Camel White	N/A	N/A	N/A	N/A	N/A	3	N/A	N/A	N/A	N/A	7	4000.00	574.49	610.17	1184.66	12	4347.83	568.60	663.23	1231.83	57	5134.31	549.37	783.20	1332.57
	Total number of observations	N/A					17					82					259					280				
Zambia (ZMW)	Guards Green	N/A	N/A	N/A	N/A	N/A	3	4.89	4.69	0.67	5.37	14	4.45	4.80	0.61	5.41	8	4.19	4.70	0.58	5.28	N/A	N/A	N/A	N/A	N/A
	Safari Menthol	N/A	N/A	N/A	N/A	N/A	4	8.52	4.69	1.17	5.87	5	5.00	4.80	0.69	5.49	47	4.64	4.70	0.64	5.34	N/A	N/A	N/A	N/A	N/A
	Peter Stuyvesant Filter	N/A	N/A	N/A	N/A	N/A	2	19.18	4.69	2.65	7.34	6	18.00	4.80	2.48	7.28	19	7.91	4.70	1.09	5.79	N/A	N/A	N/A	N/A	N/A
	Camel Blue	N/A	N/A	N/A	N/A	N/A	3	N/A	N/A	N/A	N/A	33	21.49	4.80	2.96	7.76	7	20.46	4.70	2.82	7.52	N/A	N/A	N/A	N/A	N/A
	Dunhill Switch	N/A	N/A	N/A	N/A	N/A	4	31.97	4.69	4.41	9.10	7	30.00	4.80	4.14	8.94	18	27.90	4.70	3.85	8.55	N/A	N/A	N/A	N/A	N/A
	Total number of observations	N/A					14					73					135					N/A				
Zimbabwe (USD)	Remington Gold	8	0.99	0.40	0.13	0.53	73	1.01	0.40	0.13	0.54	331	1.00	0.40	0.13	0.53	48	0.54	0.23	0.07	0.30	9	0.21	0.03	0.06	0.08
	Ascot Toasted	28	0.99	0.40	0.13	0.53	490	1.01	0.40	0.13	0.54	70	1.00	0.40	0.13	0.53	30	1.36	0.23	0.18	0.40	4	N/A	N/A	N/A	N/A
	Pacific Blue	14	1.26	0.40	0.16	0.56	511	1.06	0.40	0.14	0.54	1147	1.55	0.40	0.20	0.60	371	0.54	0.23	0.07	0.30	10	0.19	0.03	0.05	0.08
	Madison Toasted	15	1.49	0.40	0.19	0.59	110	1.51	0.40	0.20	0.60	83	2.50	0.40	0.33	0.73	11	1.81	0.23	0.24	0.46	64	0.19	0.03	0.05	0.08
	Branson Mint	22	1.79	0.40	0.23	0.63	57	1.82	0.40	0.24	0.64	1156	2.00	0.40	0.26	0.66	358	0.54	0.23	0.07	0.30	60	0.17	0.03	0.05	0.07
	Newbury Filter	16	1.79	0.40	0.23	0.63	64	1.97	0.40	0.26	0.66	78	2.00	0.40	0.26	0.66	21	2.26	0.23	0.29	0.52	13	0.26	0.03	0.07	0.10
	Total number of observations	130					1541					3243					883					197				

Appendix table IV: Year-to-year change in nominal price (2016-2020), by brand category, for single-sticks

Countries*	Brand Category	Brand Names	Reported Currency	Nominal prices				
				2016	2017	2018	2019	2020
Botswana	Value	Peter Stuyvesant Blue	BWP	3	2.5	2.5	2.5	2.5
	Popular	Craven A Menthol		3	2.5	2.5	2.5	2.5
	Premium	Dunhill Menthol		3.5	3	3	2.5	2.5
Ethiopia	Value	Nyala Filter	ETB	N/A	N/A	1	1	1
	Popular	Rothmans Blue		N/A	N/A	6	6	5
	Premium	Marlboro Red		N/A	N/A	5	5	5
Lesotho	Value	Sun White	LSL	1.5	1	1	1.5	1.5
	Popular	Peter Stuyvesant Filter		2.5	2.5	2.5	3	3
	Premium	Dunhill Courtleigh		3	3	3	3	3
Madagascar	Value	News Red	MGA	N/A	N/A	N/A	100	150
	Popular	MÃ©Lia		N/A	N/A	N/A	160	200
	Premium	Good Look		N/A	N/A	N/A	200	250
Mozambique	Value	Caesar Menthol	MZN	N/A	N/A	N/A	5	2.5
	Popular	Pall Mall Blue		N/A	N/A	N/A	5	5
	Premium	Dunhill Double Capsule		N/A	N/A	N/A	10	10
Namibia	Value	Aspen	NAD	2	2	1.5	4	N/A
	Popular	Pall Mall Blue		2	2	2	2.5	N/A
	Premium	Dunhill Kingsize		3	3	3	4	N/A
Nigeria	Value	Oris	NGN	N/A	N/A	10	10	10
	Popular	Dorchester Menthol		N/A	N/A	10	20	15

	Premium	Dunhill Switch		N/A	N/A	10	30	35
South Africa	Value	Rg Blue	ZAR	1	0.5	1	1.5	2
	Popular	Pall Mall Blue		2	1.5	1.5	2.5	2
	Premium	Dunhill Courtleigh		2.5	3	3	3.5	3.5
Tanzania	Value	Master Filter	TZS	N/A	100	150	150	200
	Popular	Club Filter		N/A	100	150	150	200
	Premium	Embassy		N/A	200	250	250	300
Zambia	Value	Guards Red	ZMW	N/A	N/A	0.5	0.5	N/A
	Popular	Life Menthol		N/A	N/A	0.5	0.25	N/A
	Premium	Peter Stuyvesant Filter		N/A	N/A	1	0.5	N/A
Zimbabwe	Value	Pacific Blue	USD	0.05	0.05	0.05	0.1	N/A
	Popular	Madison Toasted		0.1	0.1	0.1	0.2	N/A
	Premium	Kingsgate		0.1	0.1	0.1	0.15	N/A

Source: Authors’ own calculation using the database of ACP Project and WHO’s Global Tobacco Epidemic Report

Price comparison between GTR and ACP project

Table 4 presents a comparison between the GTR prices of the most sold brands with those from the ACP data. All the prices were converted to USD using the conversion rate for that year. In some countries the GTR prices were similar to the price of the premium brands in ACP or higher, for example, Namibia, Lesotho, and Nigeria, while in others they are closer to prices of value brands such as in Tanzania, Malawi, Mozambique, Madagascar, and Ethiopia. There is also a contrast in the tax pass-through estimates between GTR and ACP prices, where they are overshifted in the former, in the ACP data they are mostly undershifted.

Appendix table V: Consistency of GTR's most sold brand with ACP prices (in USD) for Packs of 20 cigarettes

Countries	GTR 2016	ACP 2016			GTR 2018	ACP 2018			GTR 2020	ACP 2020		
		Value	Popular	Premium		Value	Popular	Premium		Value	Popular	Premium
Botswana	3.1	2	3.1	3.2	3.8	2.7	3.8	4.2	4.3	2.5	3.9	4.7
Ethiopia	---	---	---	---	---	---	---	---	1.2	0.7	2	3.3
Lesotho	---	---	---	---	3.1	0.8	2.8	2.9	2.8	1.3	2.6	2.7
Madagascar	---	---	---	---	---	---	---	---	1	0.8	1.4	2
Malawi	---	---	---	---	---	---	---	---	0.7	0.7	2.2	2.3
Mozambique	---	---	---	---	---	---	---	---	0.8	1.3	2.4	2.6
Namibia	3.1	1.2	1.4	2.4	3.8	1.7	2.3	3.3	3.6	1.7	1.8	3.2
Nigeria	---	---	---	---	---	---	---	---	1	0.4	0.8	0.8
South Africa	2.5	1.3	2.2	2.6	2.9	1.5	3	3.1	2.6	1.6	2.8	3
Tanzania	---	---	---	---	1.5	1.5	1.7	1.8	1.7	2.2	2.2	2.6
Zambia	---	---	---	---	1.8	0.4	0.9	3	---	---	---	---

Zimbabwe	1.8	0.9	1.5	2	1.8	1	2	2	1.2	0.6	0.7	1.5

Source: Authors' own calculation using the database of ACP Project and WHO's Global Tobacco Epidemic Report
